# Targeted Simulation-based Leadership Training for Trauma Team Leaders

**DOI:** 10.5811/westjem.2019.2.41405

**Published:** 2019-04-16

**Authors:** Elizabeth D. Rosenman, Marie C. Vrablik, Sarah M. Brolliar, Anne K. Chipman, Rosemarie Fernandez

**Affiliations:** * University of Washington School of Medicine, Department of Emergency Medicine, Seattle, Washington; † University of Florida College of Medicine, Department of Emergency Medicine, Gainesville, Florida

## Abstract

**Introduction:**

Effective team leadership is linked to better teamwork, which in turn is believed to improve patient care. Simulation-based training provides a mechanism to develop effective leadership behaviors. Traditionally, healthcare curricula have included leadership as a small component of broader teamwork training, with very few examples of leadership-focused curricula. The objective of this work is to describe a novel simulation-based team leadership curriculum that easily adapts to individual learners.

**Methods:**

We created a simulation-based team leadership training for trauma team leaders in graduate medical education. Participants included second- and third-year emergency medicine and surgery residents. Training consisted of a single, four-hour session and included facilitated discussion of trauma leadership skills, a brief didactic session integrating leadership behaviors into Advanced Trauma Life Support®, and a series of simulations and debriefing sessions. The simulations contained adaptable components that facilitated individualized learning while delivering set curricular content. A survey evaluation was administered 7–24 months following the training to assess self-reported implementation of trained material.

**Results:**

A total of 36 residents participated in the training and 23 (64%) responded to the survey. The majority of respondents (n = 22, 96%) felt the training was a valuable component of their residency education and all respondents reported ongoing use of at least one behavior learned during the training. The most commonly cited skills for ongoing use included the pre-arrival brief (n = 21, 91%) and prioritization (n = 21, 91%).

**Conclusion:**

We delivered a leadership-focused, simulation-based training that 1) adapted to learners’ individual needs, and 2) was perceived to impact practice up to 24 months post-training. More work is needed to understand the impact of this training on learner knowledge and behavior, as well as patient outcomes.

## INTRODUCTION

Leadership is important in healthcare resuscitation teams, such as trauma teams, that function under complex, dynamic, and time-pressured conditions.^1^,^2^ Effective team leadership is linked to better teamwork,^3^ which in turn is believed to improve patient care.^4^ Despite consensus on the importance of leadership training, clinical team leadership is most frequently a small component of broader teamwork-focused training, with very few examples of leadership-focused curricula.^5^ As a result, leadership skills can vary markedly within a cohort of trainees.

Simulation-based training provides a mechanism to develop effective leadership behaviors. However, structured implementation of a context-specific leadership curriculum, such as a trauma leadership curriculum, requires 1) authentic reproduction of the environmental components present during a trauma resuscitation, 2) re-creation of a large, multidisciplinary team with scripted roles, and 3) the ability to address individual learner needs.

To address this gap in training practices, we designed a simulation-based, trauma team leadership curriculum intended for graduate medical education. This approach was novel in its use of simulation to individualize training in a dynamic setting. The objective of this article is to describe the team leadership curriculum. We also present a self-report of trained leadership skill implementation 7–24 months following training.

## METHODS

### Overview

We designed and implemented a novel, simulation-based team leadership training for trauma team leaders. The training was administered monthly, from June 2016–November 2017. We surveyed trained participants 7–24 months following training to determine the perceived value of this training. The institutional review board at the University of Washington approved the study.

### Participants and Setting

Participants included second- and third-year emergency medicine (EM) and surgery residents rotating as the trauma team leader at a Level 1 trauma center within an academic healthcare system. To be eligible, the participants were required to a) be in good standing with the Office of Graduate Medical Education, b) have completed the Advanced Trauma Life Support^®^ (ATLS) course, and c) have at least four weeks prior experience in emergency department (ED) trauma care. Residents were approached and consented by a study coordinator. Participation was voluntary and participants were compensated with a $100 gift card for study participation. Leadership training took place at the WWAMI (Washington, Wyoming, Alaska, Montana, and Idaho) Institute for Simulation in Healthcare, an 8,000-foot simulation suite, using a SimMan^®^ human patient simulator (Laerdal Medical, Wappingers Falls, New York).

### Leadership Behaviors

A conceptual model for team leadership provided the foundation for the training.^6^ Leadership behaviors were translated into “communication events” tightly linked to key time points during an ATLS-driven resuscitation. These communication events became the behaviors that were the focus of training. By linking leadership concepts (e.g., setting priorities) to key steps in ATLS, the training helped learners anchor new behaviors on an existing knowledge scaffolding. The learning objectives, organized by communication event, are provided in Table 1.

### Training Design and Implementation

Training consisted of a single, four-hour session and included a group discussion, a brief lecture, and a series of simulations and debriefing sessions. Each training session was delivered to a pair of learners. A core group of four emergency physicians and one emergency nurse served as instructors, with at least three instructors present at every session. Prior to training implementation, we piloted the curriculum with four EM residents over two sessions, using participant feedback to make minor adjustments to the timing and the content.

#### Facilitated Discussion and Lecture

The training session started with a brief introduction followed by a 30–45 minute facilitated discussion in which the learners discussed examples of effective and ineffective team leadership that they had observed in the clinical setting. Through this discussion, learners generated a list of desirable leadership behaviors and a list of barriers to implementing effective leadership. Following this discussion there was a 30-minute lecture reviewing the leadership behaviors as outlined in the learning objectives in Table 1. Learners were also provided a leadership checklist created from the learning objectives (Supplemental Figure 1). This checklist was used to facilitate learner observations during simulations and peer-to-peer feedback during debriefing.

#### Simulation Scenarios

Four trauma-resuscitation simulation scenarios were created by two members of the research team (EDR, RF) and reviewed by two additional members of the research team (Marie C. Vrablik, Anne K. Chipman) for content and flow. Scenarios were designed using event-based training design principles, which uses embedded triggers to ensure case progression regardless of learner performance.^8^

To facilitate transfer of learned behaviors to the clinical environment, we optimized the realism of the simulated environment, specifically focusing on the environmental and team factors that can contribute to the stress of leading a trauma resuscitation team. To reproduce the noise typical of an ED, actual background sound from the ED, without identifying information, was used. This sound was played during simulated cases. We recruited hospital volunteers to function as team members, allowing us to have up to 10 people representing different disciplines, in the simulation space. We introduced interruptions such as overhead pages and phone calls from nursing staff taking care of other patients. We were also cognizant of the impact of space on team interactions, and physically enclosed an area within the simulation suite to match the dimensions of the clinical environment at our institution. All four scenarios involved trauma patients presenting to the ED via emergency medical services. A detailed description of the scenarios is provided in the Supplemental Figure 2.

#### Observation and Debriefing

During the simulation component, a single learner functioned as the team leader, while the second learner observed using the leadership checklist. Debriefing occurred immediately following the simulation, and the learner in the observer role was encouraged to actively participate. The learners switched roles for the second simulation. Following the first round of simulations and debriefings, both learners identified three specific behaviors to work on in the subsequent simulations. These self-identified areas for improvement, as well as observations made by the instructors, informed the content of the subsequent simulations. The second round of simulations was structured similarly, with each participant functioning as the team leader once, and as an observer once, with a group debriefing after each scenario. The three previously identified behaviors were specifically reviewed and a plan created for each learner to facilitate implementation of these behaviors in the clinical environment.

#### Adaptive Simulation Component

The initial simulation provided a basic platform to allow learners to perform trained leadership behaviors. As noted above, the second simulation was individualized, based on learner- and instructor-identified weaknesses raised during the initial debriefing. To accomplish this we started with a scenario scaffold, and then selected from several pre-scripted options related to content (e.g., different injuries), team roles (e.g., disruptive team member), and environmental stimuli (e.g., distractions from other patients) (Supplemental Table). For example, if a learner needed to practice handing off leadership, the scenario would be modified to ensure there was a procedure (e.g., intubation, chest tube) and the team was instructed to not perform the procedure (e.g., the team “intern” would state he/she was not trained on that procedure yet), thereby forcing the team leader to do the procedure. Finally, if needed, a team member would prompt leadership hand-off if the learner did not initiate it (e.g., the “attending” would enter the room and would use escalating prompts to ensure the learner eventually handed off leadership).

### Evaluation

We developed an 18-item survey to evaluate the perceived value of the training and the extent to which trained leadership behaviors were implemented following training (Supplemental Figure 3). The survey included demographic questions, followed by multiple-choice questions related to the quality of the training, the relative value of the training compared to other leadership trainings, the realism of the simulations, and the frequency with which participants used and/or taught the learned behaviors. In addition, three questions allowed participants to provide free-text commentary on the value of the training and recommendations for improving the training. The survey was administered at the end of the academic year after the last training session was conducted. We sent a total of four email requests on a weekly basis to encourage participation. We collected data using REDCap (Research Electronic Data Capture) tools hosted at the University of Washington’s Institute of Translational Health Sciences.^9^ Surveys were anonymous.

### Data Analysis

We computed descriptive statistics (median and interquartile range as appropriate) for results from the Likert questions.

## RESULTS

A total of 36 residents completed the training, with one participant only completing half of the training due to a scheduling conflict. The survey was sent to all 36 participants, and 23 (64%) responded. The demographic composition of the participants who responded to the survey was similar to the larger group of trained participants (Table 2).

The majority of respondents felt the training was a “very valuable” component of their residency education (n=20, 87%) and “very valuable” to their current practice (n=16, 70%). The remaining respondents felt the training was either “valuable” or “fairly valuable,” with no respondents reporting it as “not valuable” or only “slightly valuable.” Table 3 provides the median responses for questions related to the value and the realism of the training.

All respondents indicated some ongoing use of the skills learned during the training, with the majority using the skills “daily” (n=6, 26%) or “several times weekly” (n=10, 43%). The most commonly cited skills for ongoing use included the pre-arrival brief (n=21, 91%) and prioritization (n=21, 91%) (Figure). The most commonly cited skills that participants taught to others included the pre-brief (n=20, 87%) and the huddle (n=18, 78%).

A total of 20 respondents (87%) answered one or more of the free-response questions. The majority of respondents (n=13, 50%) felt the training should be a standard, or even mandatory, part of the residency curriculum. Several components of the training were identified as being useful, including the following: 1) small groups of learners; 2) the realism of the training environment; 3) the focus on non-clinical skills; and 4) cycling between simulation and debriefing. The most common suggestion for improving the training was to offer similar training opportunities more frequently. Other suggestions included allowing participants to review their own videos, and providing follow-up coaching in the clinical environment. Table 4 has examples of responses organized by theme.

## DISCUSSION

We created a four-hour, simulation-based team leadership training for trauma team leaders in graduate medical education. Survey results showed nearly universal support for the training program. We acknowledge that the 36% of participants who did not respond to the survey may have viewed the training less favorably. The time interval between training and the evaluation ranged from 7–24 months. This timing meant all participants had completed at least two years of postgraduate training, with some respondents having completed residency training. Despite this time interval, and the concurrent learning opportunities, the evaluations suggest the training had a meaningful and lasting impact for most learners.

We believe the strength of the training was the learner-focused content, facilitated by small groups of learners and cycling between multiple simulations and debriefings. The facilitated discussion provided instructors with insight into the participants’ baseline level of knowledge and their individual challenges. The initial simulations were targeted to the learner based on prior knowledge of the learner and the facilitated discussion. Finally, the pre-scripted options for the second round of simulations facilitated rapid scenario modification to individualize the training. Together, this structure provides a semi-standardized approach to delivering team leadership training that can adapt to the learner.

Although not cited by the learners, we felt there was also value in incorporating peer observations and feedback, and using cognitive aids to help maintain focus on team leadership rather than clinical care. We were initially uncertain whether our learners would feel comfortable discussing their perceived strengths and challenges, or participating in peer-to-peer feedback. Ultimately, the addition of peer observers and peer-guided feedback was beneficial, allowing learners to observe strengths and weaknesses of different leadership styles exhibited by their peers. Furthermore, it augmented the realism of the scenarios by adding an element of stress that replicated the stress of performing in a crowded resuscitation bay.

Several participants suggested the training should be provided early in residency (eg, beginning of second year, prior to leading a code). However, determining the “right” time to administer this type of training is complicated. It was our impression that more senior residents, who were more clinically confident, seemed to get more immediate benefit out of the training. They were less likely to get distracted by the medicine, allowing them to focus more on leadership skills. It may be, however, that more junior learners actually achieve more long-term value from this type of training, even if it isn’t immediately apparent in the simulation lab. There is an argument for introducing teamwork and team leadership training earlier in medical education,^10^ rather than waiting to introduce leadership skills until the individual is already in a formal leadership role. Learning good habits from the start may be better than trying to add or modify them later.

## LIMITATIONS

There are several limitations to this work. Most importantly, the evaluation of the training was limited to learner perception, which is a level one outcome in Kirkpatrick’s framework.^11^ Further work is needed to determine the impact of this training, however well received, on learner knowledge, behavioral change, and clinical care. In addition, our response rate was 64%. We intentionally delayed survey assessments to gain learner insight into skill implementation, knowing the trade-off would be a decrease in response rate. While this response rate is within the range of previously reported response rates for surveys of phyisicians,^12^,^13^ it introduces the possibility of a selection bias favoring the training.

## CONCLUSION

We designed and implemented an adaptable, simulation-based team leadership training that had a lasting impact on the learners, as demonstrated by participant survey responses up to two years post-training. Given the resource-intensive nature of the training, more work is needed to understand the impact of this training on learner knowledge and behavior, and patient outcomes, and to understand the optimal timing for delivery of the training.

## Supplementary Information









## Figures and Tables

**Figure:**
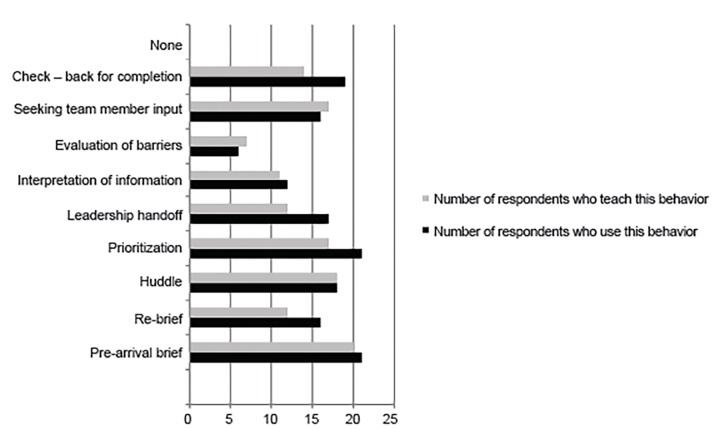
Most frequently implemented behaviors from the trauma team leadership curriculum.

**Table 1:** Learning objectives for the trauma team leadership curriculum.

Communication event	Event description	Specific team leader behaviors^a^
Assumes leadership	Prior to or upon patient arrival	•Explicit statement of role as team leader
Pre-arrival brief	Information exchange prior to arrival that occurs at the bedside to facilitate interprofessional and interdisciplinary involvement.	•Summarizing facts •Assigning or confirming roles •Creating and verbalizing a plan •Setting and verbalizing priorities
Arrival brief	Information exchange just following patient arrival and pre-hospital report to confirm or change plan as indicated.	•Highlighting any new information learned upon patient presentation •Highlighting any change in plan based on patient presentation
Huddle	Information exchange to update team as indicated during the resuscitation. Potential times include after: the primary survey, the secondary survey, a change in clinical status, or a change in team composition.	•Updating the team with summary of facts •Gathering information from team •Verbalizing primary diagnosis or problem •Setting and verbalizing priorities for next steps •Soliciting ideas from team •Asking about potential barriers or delays
Communication between services	Occurs throughout the resuscitation when new team members arrive or consultants are called.	•Updating new team members •Using SBAR^b^ to communicate •Promoting a shared mental model by explicitly asking team members and consultants to share decision making
Transferring leadership	Facilitating the hand-off of leadership to a team member when initial team member must leave the room, or engage in a procedure or other task that requires focus on a subset of the patient’s care.	•Handing off leadership when performing a procedure or leaving patient care area •Using SBAR^b^ to inform new team leader •Making transfer of leadership explicit to entire team

a Team leader behaviors are based on a conceptual model of team leadership.^6^

b Situation, background, assessment, recommendation (SBAR) is a component of the TeamSTEPPS® training program.^7^

**Table 2:** Demographics of training participants and training evaluation survey respondents.

	All participants* (n=36) N(%)	Survey respondents (n=23) N (%)
Gender		
Male	22 (61)	14 (61)
Female	14 (39)	9 (39)
PGY during training		
2	23 (64)	14 (61)
3	13 (36)	9 (39)
PGY at time of survey*		
2		6 (26)
3		9 (39)
4		6 (26)
5		0 (0)
Fellow or attending		2 (9)
Ethnicity		
Hispanic	2 (6)	2 (9)
Not Hispanic	34 (94)	20 (87)
Unknown or not reported		1 (4)
Race		
AN/AI	0 (0)	0 (0)
Asian	7 (19)	3 (13)
Native Hawaiian or Pacific Islander	0 (0)	0 (0)
Black	0 (0)	0 (0)
White	27 (75)	18 (78)
More than one	2 (6)	2 (9)
Specialty		
Emergency medicine	31 (86)	20 (87)
Surgery	5 (14)	3 (13)

*PGY*, post-graduate year; *AN*, Alaskan Native; *AI*, American Indian.

* The All Participant data was taken from the demographic survey completed by participants at the time of training with the exception of the “PGY at the time of survey,” which was only available for those participants who responded to the follow-up survey.

**Table 3:** Survey results for the perceived value and realism of the simulation-based leadership training.

Question and anchors	Median score (IQR)
Value of training to residency education 1 - Not valuable, residency training should not include this training 3 - Fairly valuable, residents in my specialty should have the option of taking this training 5 - Very valuable, it should be a part of all residency programs in my specialty	5 (5,5)
Value of training to current practice 1 - Not valuable, it was much less impactful than other teamwork or leadership training 3 - Fairly valuable, it was as impactful as other teamwork or leadership training 5 - Very valulable, it was more impactful than any other leadership or teamwork training	5 (4,5)
Realism of the simulations 1 - Not realistic, the simulation did not represent the stress and environment present in a trauma resuscitation 3 - Fairly realistic, some elements of the stress and environment of a trauma resuscitation were well-represented 5 - Very realistic, the stress and environment of a trauma resuscitation were well-represented	4 (4,5)

*IQR*, interquartile range.

**Table 4:** Examples of survey free-text responses organized by themes.

Themes (Number of related comments)	Examples of comments
Training should be required (13)	Should be mandatory. Please incorporate this in our training! It is one of the single most helpful things I have done in residency regarding leadership. This has absolutely changed my practice. Mandatory for all residents before leading a code. Excellent training which gives a framework for myriad roles in daily clinical medicine. Should be a component of every resident’s training. Excellent, should be provided to all EM residents in all residency programs, it helps the quality of care in our specialty.
Useful components of the training	
Realism of the simulations (SIM) (3)	This was very helpful, and far more realistic than the average SIM. It would be valuable for all residents to receive this training! The authenticity and stressful environment made this great training.
Non-clinical focus (1)	This was one of the most valuable simulations I participated in and made me a much more confident leader in these situations. Prior to the simulation I was a bit of a wallflower but this gave me some basics with which to take command and fall back on in difficult situations. Rather than focusing on the basics of resuscitation the emphasis on teamwork was key. I recently had a very difficult code and was able to take command with many of the specific skills that I learned in this training.
Small learner group (1)	Nothing to make it better, but the very small group (two people) was very helpful.
Repetition of simulations and debriefings (1)	Opportunity to do multiple SIMs after discussing how the first one went, and getting a second chance to incorporate the teachings.
Opportunity for improvement	
Coaching and performance review (5)	Ability to see feedback videos. Real-time feedback in a real clinical scenario. More check-ins after the training to see how things were going.
More frequent training (4)	We need more of this kind of training. One day spent doing this training drastically changed my performance during traumas and medics codes and really helped with my confidence. More of it. More repetitions.
Timing of training (4)	If it had happened earlier in my training, at the end of R1 or beginning of R2, before I had certain set habits. Ideal for junior residents to set them on the correct path.
Other (6)	Critical training, not covered elsewhere. Invaluable. Made me a better team leader.

*EM*, emergency medicine; *R1*, first-year resident; *R2*, second-year resident.
