# Supplementary material for: Targeted Simulation-based Leadership Training for Trauma Team Leaders
Source: West J Emerg Med. 2019 Apr 16;20(3):520–6. doi: 10.5811/westjem.2019.2.41405 (PMC6526881; doi:10.5811/westjem.2019.2.41405)
Supplement: Supplementary file 1 [file wjem-20-520-s001.docx]

**Supplemental Figure 1**. Leadership Checklist Used To Facilitate Peer-to-Peer Observations and Feedback

**Observer Instructions**: Your job is to observe your colleague during a simulated trauma resuscitation and complete the following checklist. You will participate in the debriefing following the scenario. Please look over the checklist in advance to remind yourself of the behaviors you are looking for. The checklist is meant to guide feedback and is not something you, or your colleague, will be graded on. You are welcome to take notes. REMEMBER TO FOCUS ON LEADERSHIP BEHAVIORS, NOT THE CLINICAL CARE.

**Primary survey.** Pay attention to if and how the TL “huddles” after a critical or abnormal finding.

**Surgery team arrives.** How does the TL respond to the new arrivals and multiple conversations? Focus on communication.

**Secondary survey.** Continue looking for “huddles.” How does the TL move from the initial evaluation to the next step (diagnostics, treatments, procedures)?

**EMS call.** Patient with concerning mechanism of injury, but stable vital signs.

**Participant enters**. EMS hand-off with change in patient condition. Pay attention to how TL responds to new information.

**BRIEF occurred: Draw an arrow to when this occurred**

- Summarized facts
- Assigned and/or confirmed roles
- Verbalized plan
- Verbalized priorities

**Comments:**

**RE-BRIEF occurred: Draw an arrow to when this occurred**

Did the timing seem appropriate?

Did it include the following:

- Confirmed patient condition **OR** Verbalized change in condition
- Reassigned roles if indicated
- Confirmed existing plan and priorities **OR** Verbalized changes

**Comments**:

**Hand-off/consultation COMMUNICATION:**

- **S**ituation (“One-liner”)
- **B**ackground
- **A**ssessment
- **R**ecommendations (Next steps)

**Comments:**

**HUDDLES: Draw an arrow to when these occurred. Some of the behaviors you may see are:**

- Summarized facts
- Verbalized primary diagnosis/problem
- Verbalized plan
- Set priorities for next steps
- Solicited ideas from team
- Asked about delays/barriers

**Comments** (e.g. was anything done exceptionally well?):

**Was there a time when a huddle would have been useful, but wasn’t performed? When?**

*EMS*, emergency medical services; *TL*, team leader.
