# Supplementary material for: Targeted Simulation-based Leadership Training for Trauma Team Leaders
Source: West J Emerg Med. 2019 Apr 16;20(3):520–6. doi: 10.5811/westjem.2019.2.41405 (PMC6526881; doi:10.5811/westjem.2019.2.41405)
Supplement: Supplementary file 2 [file wjem-20-520-s002.docx]

**Supplemental Figure 2.** Flow Sheets for Simulation Scenarios A-D.

**EMS radio call**

“30 year-old male in an MCC, a little confused – GCS 14, VS stable, ETA 2 min”

**VS trend over 10 min:**

HR = 90 →130

BP = 110/70 →75/50

O2 Sat = 98%

RR = 12 (or vent setting)

EtCo2 = 35 (only if requested)

**Time = 0 Min**

**Time = 2 Min**

**Primary survey**

A – clear, moaning

B – symmetric breath sounds

C – symmetric pulses

**RN prompts** intubation if needed

**Time = 10 Min**

**Time = 12 Min**

Available ~**2 min** after ordered:

CXR, FAST = normal

Pelvis XR = fracture

Hgb = 13.1

ABG = 7.25/35/100, (lactate 4.5)

**Time = 14 Min**

**Surgery team** “huddles,” and leaves

**OR/CT/IR calls for patient**

**Scenario A: MCC**

**Secondary survey**

**ED attending prompts** imaging if needed

**VS trend after initiation of blood products:**

HR = 130 → 110

BP = 75/50 → 95/75

**ED attending prompts** blood products and calls trauma team if needed

All images available

**Participant enters room**

Patient moaning to painful stimuli only, not answering questions, eyes closed

On back-board, c-collar

EMS available for questions

**Time = 5 Min**

**ED attending and MA enter**

ED attending prompts or performs intubation if needed

**Becomes unresponsive at ~3 min**

**Surgery team enters**

**EMS radio call**

“30 year-old male in a MVC, intubated for AMS, VS stable, ETA 2 min”

**VS trend over 10 min:**

HR = 90 →130

BP = 110/70 →75/50

O2 Sat = 98%

RR = 12 (or vent setting)

EtCo2 = 35 (only if requested)

**Time = 0 Min**

**Time = 2 Min**

**RN places IV**

**Primary survey**

A – endotracheal tube in place

B – symmetric breath sounds

C – **pulse** **absent left foot**

**RN/intern can prompt** absent pulse

**Time = 10 Min**

**Time = 12 Min**

Available ~**2 min** after ordered:

CXR, pelvis XR = normal

RLE = tibia/fibula fracture

FAST= free fluid

Hgb = 13.1

ABG = 7.25/35/100, (lactate 4.5)

**Time = 14 Min**

**Surgery team** “huddles,” and leaves

**OR/CT/IR calls for patient**

**Scenario B: MVC**

**Secondary survey**

**ED attending prompts** imaging

**VS trend after initiation of blood products:**

HR = 130 → 110

BP = 75/50 → 95/75

**ED attending prompts** blood products and calls trauma code if needed

All images available

**Participant enters room**

Patient intubated, on back-board, c-collar

EMS available for question, **IV not working**

**Time = 5 Min**

**Pulses return with traction and/or splinting**

**ED attending and MA enter**

**Surgery team enters**

**VS trend over 5 min:**

HR = 90 → 130

BP = 110/70 → 75/50

O2 Sat = 98%

RR = 12 (or vent setting)

**Time = 0 Min**

**Time = 2 Min**

**Primary survey**

A – clear, moaning

B – symmetric breath sounds

C – symmetric pulses

**RN prompts** intubation if needed

***Call from RN about another patient**

**Time = 7 Min**

**Time = 10 Min**

Available ~**2 min** after ordered:

CXR, FAST = normal

Pelvis XR = fracture

Hgb = 13.1

ABG = 7.25/35/100, (lactate 4.5)

**Time = 12 Min**

**Surgery team** enters the room

**Scenario C: Fall From Tree**

**Secondary survey**

**Dilated right pupil**

**RN prompts** imaging if needed

**VS trend after initiation of blood products:**

No change

**RN prompts** blood products if needed

All images available

**Time = 5 Min**

**Attending handoff**

**(*EMS handoff if arrival brief needed)**

Patient is a 50 year-old male who fell out of a tree, “stable” in transport, now moaning

On back-board, c-collar, pelvis sheeted

**Clinical problems**

- Blown right pupil
- Pelvis sheeted incorrectly
- Pelvic fracture
- Other patients

***Prioritizing challenge**

**ED attending** intubates if asked *Can make team leader do it for a **handoff opportunity**

**VS trend over 5 min**

**(start when intubated):**

HR = 90 → 130

BP = 110/70 → 75/50

O2 = 95% → 85%

RR = 12 (or vent setting)

*pupil change ~3 min

**Time = 0 Min**

**Time = 2 Min**

**Primary survey**

A – clear, intubated

B – symmetric breath sounds

C – symmetric pulses

**Time = 5 Min**

**Time = 7 Min**

Available ~**2 min** after ordered:

Pelvis XR, FAST = negative

CXR = tension PTX

Hgb = 13.1

ABG = 7.25/35/100, (lactate 4.5)

**Time = 10 Min**

**Surgery team enters**

**Secondary survey**

***Dilated right pupil**

**ED attending prompts** imaging

**VS trend after needle decompression:**

HR = 130 → 90

BP = 75/50 → 115/85

O2 sat = 85% → 95%

**Radiology calls** with PTX findings to prompt needle decompression

**RN prompts** trauma code activation

All images available

**ED attending or other team member** does chest tube placement if requested

*Can make team leader do it for handoff opportunity

**Scenario D: Assault**

**Attending handoff**

**(*EMS handoff if arrival brief needed)**

Patient is a 25 year-old male, assaulted outside of a bar confused at the scene

Intubated pre-hospital, VS “stable”

Back-board, c-collar, pelvis sheeted

**Clinical problems:**

- Blown right pupil
- Intubated
- Tension PTX
- Other patients

***Prioritizing challenge**

Abbreviations: ABG, arterial blood gas; AMS, altered mental status; BP, blood pressure; c-collar, cervical collar; CT, computed tomography; CXR, chest x-ray; EMS, emergency medical services; ETA, estimated time of arrival; EtCO_2_, end-tidal carbon dioxide; FAST, focused assessment with sonography in trauma; GCS, Glasgow coma score; Hgb, hemoglobin; HR, heart rate; IR, interventional radiology; IV, intravenous line; MA, medical assistant; MCC, motorcycle collision; MVC, motor vehicle collision; OR, operating room; O2 Sat, oxygen saturation; PTX, pneumothorax; RLE, right lower extremity; RN, registered nurse; RR, respiratory rate; VS, vital signs
