# Supplementary material for: Targeted Simulation-based Leadership Training for Trauma Team Leaders
Source: West J Emerg Med. 2019 Apr 16;20(3):520–6. doi: 10.5811/westjem.2019.2.41405 (PMC6526881; doi:10.5811/westjem.2019.2.41405)
Supplement: Supplementary file 3 [file wjem-20-520-s003.docx]

**Supplemental File Figure 3.** Post-Training Survey.

| What is your gender? | - Male - Female |
| --- | --- |
| What was your PGY at the time of your leadership training? | - 2 - 3 - 4 - 5 |
| What was your PGY on 06/01/2018 | - 2 - 3 - 4 - 5 - Fellow or attending |
| What is your ethnicity? | - Hispanic or Latino - Not Hispanic or Latino - Unknown / Do not wish to report |
| What is your race? | - Alaskan Native / American Indian - Asian - Native Hawaiian or Other Pacific Islander - Black or African American - White - More than one - Unknown / Do not wish to report |
| What is your age? | _________________ |
| What is your specialty? | - General surgery - Emergency medicine - Other, surgical |
| What was your institution at the time of your leadership training? | - University of Washington - Madigan Army Medical Center - Other |
| In your current role, how often do you lead/run a resuscitation (medical or trauma)? Average over a month to provide an estimate. | - Very frequently (daily) - Frequently (several/week) - Occasionally (weekly) - Rarely (1-2/month) - Very rarely (<1/month) - Never (0) |
| Reflecting back on the leadership training, did you find it a valuable component of your residency education? | - Very valuable, it should be a part of all residency programs in my specialty - Valuable - Fairly valuable, residents in my specialty should have the option of taking this training - Slightly valuable - Not valuable, residency training should not include this training |
| How would you rate the realism of the simulation component of this training? | - Very realistic, the stress and environment of a trauma resuscitation were well-represented - Realistic - Fairly realistic, some elements of the stress and environment of a trauma resuscitation were well-represented - Slightly realistic - Not realistic, the simulation did not represent the stress and environment present in a trauma resuscitation - I don’t remember |
| Compared to other teamwork or leadership-focused training, how valuable did you find the training to your current practice? | - Very valuable, it was more impactful than any other leadership or teamwork training - Valuable - Fairly valuable, it was as impactful as other teamwork or leadership training - Slightly valuable - Not valuable, it was much less impactful than other teamwork or leadership training |
| Which skills/behaviors acquired during the leadership training do you incorporate in your current practice?  (check all that apply) | - Pre-brief - Re-brief - Summary brief - Prioritization - Leadership handoff - Interpretation of information - Evaluation of task barriers - Seeking team member input - Check-back for task completion - None |
| In your current practice, how frequently do you use skills learned in the leadership training? | - Very frequently (daily) - Frequently (several/week) - Occasionally (weekly) - Rarely (1-2/month) - Very rarely (<1/month) - Never (0) |
| Which skills acquired during the leadership training do you believe you are teaching or transferring to new learners?  (check all that apply) | - Pre-brief - Re-brief - Summary brief - Prioritization - Leadership handoff - Interpretation of information - Evaluation of task barriers - Seeking team member input - Check-back for task completion - None - I don’t teach in my current role |
| What would you say to a program director or administrator about this training? | ____________________________________________ |
| Reflecting back, what would have made this training better? | ____________________________________________ |
| Please provide any additional comments. | ____________________________________________ |
