# Supplementary material for: Targeted Simulation-based Leadership Training for Trauma Team Leaders
Source: West J Emerg Med. 2019 Apr 16;20(3):520–6. doi: 10.5811/westjem.2019.2.41405 (PMC6526881; doi:10.5811/westjem.2019.2.41405)
Supplement: Supplementary file 4 [file wjem-20-520-s004.docx]

**Supplemental File Table.** Optional Adaptations for Scenarios C and D to Facilitate Individualized Leadership Training

| **Targeted Events and Behaviors** | **Adaptations** |
| --- | --- |
| Pre-arrival brief and/or arrival brief | - Have pre-hospital radio call similar to scenarios A and B, rather than a hand-off from the attending physician. |
| Leadership hand-off | - ED attending declines to intubate, insisting team leader performs the intubation (scenario C) - Junior team member indicates he/she is not comfortable performing a tube thoracostomy, and ED attending delays returning until after procedure is performed (scenario D) |
| Prioritization | - Instructor calls in one or more times as a nurse with questions regarding other patients - Overhead page for the team leader for a new patient - Team identifies additional injuries (e.g., positive FAST, pulseless extremity) |
| Huddle | One or more members of the team become challenging until team leader provides an update:   - Primary nurse – e.g., can prepare patient for operating room, start hanging blood, etc., prior to determination of injuries - Team members – e.g., start socializing, talking to each other, talking over the team leader - Senior physicians – e.g., ED and surgery attendings can communicate primarily with each other, excluding the team leader from updates and decision making   *Can be scaled to team leader response - e.g., team cooperation improves following a huddle in which the team member shared his/her decision making and sought input from team members |
| Communication Between Services - SBAR | - Instructor calls in as consultants from various services prompting the team leader to communicate using the SBAR technique multiple times on the same patient, adjusting the content to the desired response from that consultant |

*ED*, emergency department; *FAST,* focused assessment with ultrasonography in trauma; *SBAR,* situation, background, assessment, recommendation.
